# A Review of Parent-Implemented Early Start Denver Model for Children with Autism Spectrum Disorder

**DOI:** 10.3390/children9020285

**Published:** 2022-02-18

**Authors:** Rong-An Jhuo, Szu-Yin Chu

**Affiliations:** Department of Special Education, National Tsing Hua University, Hsinchu 300044, Taiwan; imjb110257@gapp.nthu.edu.tw

**Keywords:** autism spectrum disorder, fidelity, parent-implemented Early Start Denver Model

## Abstract

The purpose of this review is to provide an overview of findings from selected research published between 2012 and 2022 in English-language peer-reviewed journals to evaluate the effectiveness of the parent-implemented Early Start Denver Model (P-ESDM). Thirteen studies used quantitative methods to examine the relationship between variables. We considered four categories of variables in the analysis: child characteristics, intervention intensity and duration, child outcome measures, and parent-related outcome measures and parental fidelity. The findings revealed positive child–parent-related outcomes. In addition, the quality of implementation, relating to parent fidelity, should be considered when evaluating the efficacy of the intervention. However, only half the studies revealed that the standard benchmark for acceptable fidelity was being achieved. Implications for future research and practice are discussed.

## 1. Introduction

Autism spectrum disorder (ASD) is a neurodevelopmental disorder that can cause difficulties in communication and social interaction. Children with ASD may maintain the presence of restricted interests and repetitive behaviors [1]. Furthermore, evidence suggests that reliable diagnosis of children with ASD can be made before the age of 2 years [2]. An emphasis on very early intervention in several reviews suggests the importance of effective early intervention which may relate to greater possibility of children’s learning and progress [3]. Accordingly, early intervention is key and leads to better outcomes in shaping the child’s developing brain and is especially crucial for young children with social and developmental disabilities [4,5].

Early intervention research for ASD has increased dramatically in the past decade. Naturalistic developmental behavioral interventions (NDBIs) have been tested by clinical trials of behavioral treatments [5,6,7,8,9]. NDBIs integrate developmental and relationship-based approaches with applied behavioral analysis (ABA) strategies and are implemented in the child’s day-to-day environment, including in play and routine activities where many learning opportunities can be embedded [10,11,12]. In many interventions, natural reinforcement strategies are used which focus on the child’s choices and rewards closely related to the learning activities [13].

Among evidence-based practices, the Early Start Denver Model (ESDM) is a representative approach to NDBIs. In addition, ESDM follows comprehensive NDBI principles, grounded in developmental and behavioral science [14,15] and neuroscientific evidence [16]. Based on ABA and developmental psychology, ESDM is a comprehensive early intervention that aims to reduce the severity of ASD symptom and emphasize in the development of cognitive, social, emotional, and language abilities [4,17,18]. The model was designed for use with children aged 18–48 months and features a manualized curriculum divided into four levels [19], each of which targets different developmental areas. Moreover, by emphasizing natural environments and positive relationships to promote children’s learning outcomes, ESDM acknowledges that parents are best placed to implement early interventions in the home setting [3,18,20,21].

Grounded in Bronfenbrenner’s [22] ecological systems theory, the theoretical framework indicates that multiple levels of the child’s environment influence child development. The microsystem is the smallest system in which relationships between the child and parent are constructed [23]. Parents play a crucial role in the early interventions provided to young children with disabilities, helping foster the child’s growth and development [24]. Parenting a child with autism can be rewarding but also challenging [25,26,27]. Furthermore, research underscored that parents of children with ASD consistently reported lower levels of parenting-related anxiety, stress, or depression [5,28]. Indeed, many studies on NDBIs have focused on evaluating the effects of early intervention when delivered by parents. Recent studies have demonstrated that ESDM intervention strategies can be implemented by parents to maximize learning opportunities in daily activities and bridge service gaps; this intervention is known as parent-implemented ESDM (P-ESDM) [15,28]. Several studies have revealed that (a) parents can learn to implement the intervention techniques with fidelity and (b) doing so results in a range of improvements in child outcomes [7,8,14,29,30]. Thus, research recommended that parents can plausibly incorporate naturalistic techniques into daily routines in order to accelerate the maintenance and generalizability of treatment gains [31,32].

P-ESDM is an evidence-based structured approach that teaches parents ESDM techniques such as gaining the child’s attention and motivating them, promoting dyadic engagement and joint activity routines, enhancing verbal and nonverbal communication, and incorporating play skills [26]. To determine whether improvements in the child’s development result from corresponding changes in the techniques of parent’s interaction, one must take parent’s use of the intervention strategies into account [33]. Treatment fidelity is defined as “the methodological strategies used to monitor and enhance the reliability and validity of behavioural interventions” [34] (p. 139). The treatment fidelity is considered acceptable for an adherence of 80% or more [35]. However, the role of treatment fidelity relates to child intervention response needs further investigation [36]. Thus, measuring treatment fidelity is crucial for clarifying this relationship.

Investigating prior research in the field is valuable because it reveals the present state of scholarship and offers guidance to researchers who are seeking suitable topics to explore [37]. Because parent-implemented intervention has become increasingly popular in early interventions for children with ASD [28], fewer published reviews have focused explicitly on studies evaluating the parent fidelity of implementation. Waddington et al. [38] analyzed the effectiveness of ESDM, including in relation to parent-related outcomes and fidelity. However, the results focused primarily on intervention characteristics. To fill the gap in the literature, in this study, we sought to provide a summary of the effectiveness of ESDM adopted by parents and caregivers and further discussed parent fidelity.

## 2. Materials and Methods

### 2.1. Search Procedures

The online database Google scholar was used to search for studies that explicitly investigated the effectiveness and fidelity of P-ESDM implementation. The search was confined to English language peer-reviewed papers in a 10-year period 2012–2022 (February). The following search terms were entered to search for article titles, abstracts, or full texts: parent, parent-delivered, parent-implementation, parent-training, family, caregivers, and ESDM. Preliminary searches found 3620 articles in the database searched. Research articles were further screened using a two-step screening process. First, the authors would read the abstract and methods initially when the article was located. Research articles consisted of the ESDM delivered by parents or caregivers were assessed. In the second step, the studies which were relevant and appropriate for our focus and satisfied the inclusion and exclusion criteria mentioned below would be included for this review.

### 2.2. Inclusion and Exclusion Criteria

Research articles were included for review if they: (a) were evidenced-based studies evaluating the outcomes of an intervention involving parents or caregivers; (b) were published at any time between 2012 and 2022; (c) reported data from an empirical or behavioral intervention using experimental, quasi-experimental, or single-case designs; and (d) enrolled children with age of less than 60 months (this is the oldest recommended age for ESDM interventions). Research articles were excluded from review if they: (a) were but a further examination of a previous study that we had already included for review; or (b) lacked data on parent-related outcomes. Finally, after the inclusion and exclusion criteria were applied, 13 studies were identified for inclusion in the current study.

### 2.3. Data Extraction

The following pieces of information regarding the included research articles were recorded: (a) child characteristics (e.g., number of participating children, age, and diagnosis); (b) study design (e.g., randomized controlled trial, single-case multiple-baseline design, or quasi-experimental one-group pretest–posttest design); (c) intervention intensity and duration (i.e., hours per week of intervention and the total number of weeks); (d) child outcome measures (scores on cognitive, social, emotional, language, or adaptive behavior assessments, such as the MacArthur–Bates Communicative Development Inventory (MCDI), the Mullen Scales of Early Learning (MSEL), or the PATH curriculum checklist (CC)); and (e) parent-related outcome measures (e.g., parent’s perception, engagement, or skills) and results on parent fidelity.

### 2.4. Interrater Agreement

The first and second authors first coded all 13 research articles according to whether they met the inclusion and exclusion criteria and then recorded the aforementioned pieces of information regarding the included articles. The initial interrater agreement between both authors was 85%. Both authors then discussed any disagreements until 100% agreement was reached.

## 3. Results

ESDM is an evidence-based intervention integrating ABA principles [39]. To address the need for an evidence base that includes research methods evaluating effectiveness, we included interventional studies in this review. All 13 research articles in this study reported using a quantitative research design, including three that used a multiple-baseline across subjects design [5,40,41] and one that adopted a single-case nonconcurrent alternating-treatment design [42]. Of the group studies, one was a nonrandomized controlled trial [12], four were randomized controlled trials [15,28,43,44], and three used a quasi-experimental one-group pretest–posttest design [3,16,17].

Table 1 provide a summary of each study’s (a) child characteristics, (b) intervention intensity and duration, (c) child outcome measures, and (d) parent-related outcome measures and results on parent fidelity.

### 3.1. Child Characteristics

Of the 13 included studies, 12 provided P-ESDM intervention to a total of 326 children who had received a clinical diagnosis of ASD or were at risk of receiving an ASD diagnosis. In the study by Abouzeid et al. [17], 6 of the 10 children had received a concomitant diagnosis of global developmental delay (*n* = 5) or attention deficit disorder (*n* = 1). In Zhou et al. [12] and Estes et al. [28], neurological diseases of known genetic etiology (e.g., fragile X) were excluded. By contrast, Vismara et al. [5] included young children with fragile X syndrome, which is a genetic condition and the leading identified cause of ASD. In six studies [12,15,17,28,40,41,43,44,45], the ASD diagnosis of the participants was based on the Autism Diagnostic Observation Scale (ADOS) [46]. Three of these studies [5,12,28] included a clinical diagnosis of ASD based on the criteria from the Diagnostic and Statistical Manual of Mental Disorders (fourth or fifth edition) [1,47]. In the study by [42], participants were assessed using the Modified Checklist for Autism in Toddlers, Revision with Follow-up [48], whereas in the study by Waddington et al. [3], participants considered “at risk” of receiving an ASD diagnosis were assessed through the Social Communication Questionnaire [49]. Participants ranged in age from 12 to 59 months at the start of the intervention.

### 3.2. Intervention Intensity and Duration

In total, 12 of the 13 studies had P-ESDM training sessions ranging from 1 to 2 h per week over a 10–13-week period. Nonetheless, because neither the P-ESDM nor the community group exhibited significant changes, Zhou et al. [12] extended the duration of intervention to 26 weeks (two cycles including two ESDM assessments) to evaluate the effects.

### 3.3. Child Outcomes

The advantages of the P-ESDM intervention in children’s learning outcomes, specifically child behavioral functioning and development, social interaction and communication skills, autism severity, and diagnostic outcomes, were analyzed.

Six research articles reported measures for child behavioral functioning and development [5,12,15,40,41,43]. These measures were assessed using a variety of instruments, including the MCDI [50]; MSEL [51]; Vineland Adaptive Behavior Scales [52]; PATH CC [53]; Griffiths Development Scales, Chinese version [54,55]; and Child Behavior Rating Scale [56]. All six research articles reported significant improvements over time after the P-ESDM intervention. However, two of the three group studies [12,40,45] reported no differences in child outcomes between groups.

Eight research articles reported measures for observations of social interaction and communication skills [3,5,12,15,18,40,42,44]. These measures included expressive and receptive language, verbal and nonverbal responsiveness, spontaneous verbal utterances, imitation skills, symbolic play, social orienting, and joint attention. All the research articles reported positive results for all these outcome measures, except for research articles featuring group studies [15,44]; these group study articles reported no significant differences in measures pertaining to social and communication skills. In addition, one article [45] applied the ESDM Curriculum Checklist and the Denver Model commitment coding sheet to examine the effect of the intervention.

Three research articles [12,15,43] reported measures for autism severity and diagnostic outcomes using ADOS [46]. These studies reported negative results with no significant differences following intervention.

### 3.4. Parent-Related Outcomes and the Examination of Parent Fidelity

The advantages of P-ESDM intervention in parent-related outcomes, specifically parent satisfaction, parenting stress, coaching experience, parent engagement, parent-implemented goals, and parent fidelity, were analyzed.

Nine research articles [3,12,15,17,18,41,42,43,44] reported positive results in that most of the parents felt satisfied or agreed that they would recommend this approach to others. Moreover, these studies used questionnaires or measures that evaluated the parents’ willingness to participate in the program, such as the Treatment Acceptability Rating Form-Revised questionnaire [57] and the Intervention Evaluation Form (University of Washington, unpublished [43]).

Several research articles evaluated additional parent-related outcome measures. Two research articles [28,44] reported positive results for parenting stress based on the measures included in the Parenting Stress Index-Short Form [58] and the Questionnaire of Resources and Stress [59]. Three research articles [3,5,17] reported positive ratings for coaching experience and relationship with the trainer. Three research articles [5,40,41] reported positive results for parent engagement using the Maternal Behavior Rating Scale [56] or measures developed by the researcher [5].

Ten of the 13 studies examined parent fidelity in implementation [3,5,15,17,28,40,41,42,43,44]. The measures included the ESDM Fidelity Rating System [19] and the P-ESDM Fidelity Tool [60]. All these research articles reported improvements in interaction skills over the 12-week period of the P-ESDM. However, most of the parents in only 50% of the studies [3,40,41,42,44] achieved the standard benchmark (at least 80% at posttest) for acceptable fidelity using the ESDM.

## 4. Discussion and Recommendations for Future Research

This study is a systematic review of the literature on P-ESDM as an early intervention approach implemented by parents. This review included 13 research articles published after 2012. The studies’ results were categorized into (a) child characteristics, (b) intervention intensity and duration, (c) child outcome measures, and (d) parent-related outcomes measures and results on parent fidelity. First, the majority of the studies recruited children aged from 12 to 59 months with a clinical diagnosis or at risk of ASD. Second, the intensity and duration of the intervention was approximately 1–2 h per week over 12 weeks. Third, parent-implemented comparison studies failed to yield significant improvements in child outcomes relative to a treatment as usual (TAU) group. Finally, most studies reported positive parent-related outcomes.

Notably, the study by [5] examined the preliminary efficacy and acceptability of P-ESDM for young children with a developmental disorder other than ASD. Despite the fact that the ESDM model was designed and mostly focused on young children with ASD, Vismara et al. [5] demonstrated the efficacy of this model for children with other disorders. Since ESDM is a comprehensive approach that focus on a wide range of developmental behavior, we suggested that P-ESDM may be feasible for young children with developmental disorders other than ASD and benefit a larger population. However, these results should be explored in future research.

Generally, ESDM interventions were implemented for a 12-week period. Nevertheless, Zhou et al. [12] indicated that parent-delivered ESDM for 12 weeks increased parent interaction skills and child progress markedly but not to a significantly greater extent than community intervention. Their results illustrated that P-ESDM intervention for 26 weeks improves general developmental outcomes. However, this study did not examine parental fidelity of implementation of P-ESDM. In the future, the relationship between a longer treatment period, treatment administered by parents, and outcomes should be assessed.

With regard to child outcomes, most studies reported significant improvements, particularly in cognitive skills, language ability, imitation, attentiveness, and initiation of social contact. However, parent-implemented comparison studies did not demonstrate no group difference on the degree of improvement in child outcomes for the parent group relative to the control group. To overcome the difficult issues encountered in previous studies that may limit the effects of short-term low-intensity treatments, Rogers et al. [43] evaluated the effects of an enhanced version of a parent-implemented intervention. Nevertheless, the findings from this study revealed treatment fidelity was linked to proximal but not distal measurement. This suggests that within-group difference may limit the differential effects of the group on child progress which parents may not improve equally across all techniques during the intervention. The reasons for this variability in results are unclear, but a greater understanding of the experiences of parents might provide some insight [3].

Finally, the overall results of review suggest positive outcomes for parents who participate in parent-implemented ESDM, such as gaining access to a useful training program for the child’s learning needs [12,15,17,18,41,43,44], having lower levels of parenting stress [12,28], having increased confidence and capability [5,17,40,41], and experiencing instances of positive coaching [3,5,17]. Likewise, recent study suggested that a high quality, intensive and evidence-based practice may guide family to embed delivery across natural environment [61]. This review suggests that the P-ESDM is a promising treatment that have strong evidence behind; however, the results from most of these studies must be interpreted with caution. The authors of [62] examined whether intervention fidelity contributed to children’s response to ESDM. It revealed that most items included in fidelity checklist had an impact on learning response for children. Nevertheless, the intervention was delivered by therapist, the relations between parent-implementation fidelity and children’s outcomes may not be straightforward [37]; correlations between the use of ESDM techniques and child learning response varied across mother–child dyads. This highlights the need for further research into the preferences of parents for certain P-ESDM techniques [34].

Consulting the findings from the present study, we posit two recommendations for the provision of P-ESDM. First, in several studies [5,40,41,44], the role of telehealth-delivered interventions exhibited potential, making interventions more accessible, affordable, and easier for families to complete. The purpose of telehealth parent training is to support and equip parents with the tools to intervene and create natural moments with their child in addition to or while waiting for more intensive services to begin [63]. Crucially, since the onset of COVID-19 pandemic, a number of clinical trials pivoted to telehealth [64]. Telehealth may provide more opportunities for more people around the world. Furthermore, [65] compared two groups of telehealth-based intervention (self-directed and therapist-assisted) and revealed that both groups showed improvements. However, the therapist-assisted group demonstrated additional gains in social skills, which highlighted the potential benefit of implementation of telehealth-based intervention with therapist support. Second, fathers should be included in the P-ESDM intervention [3,28,40]; mothers tend to be the more involved in the implementation. The benefits of parent coaching for fathers of children with ASD is a key area for future research, and the focus on fathers should be tailored to reflect parental roles.

## 5. Limitations

This study has the following limitations. First, the review discussed parent treatment fidelity, and higher levels of parent treatment fidelity may lead to more positive treatment outcomes [66]. However, two of the studies lacked specific scores for fidelity, which could limit the overall scope of the studies for this review. Second, as [67] mentioned, since the intervention is delivered by parents, it is hard to evaluate the true dosage of intervention. The relationship between the dosage and outcomes may not be inferenced. Third, examined the effectiveness status of ESDM and suggested that additional research is needed to focus on ESDM intervention versus other evidence-based approaches. Large clinical trials may provide evidence to inference the superiority of ESDM comparing to other approaches. Forth, only peer-reviewed studies published between 2012 and 2022 were included. Although interrater agreement was conducted to reduce selection bias, other relevant research articles may not have been included and the data set may therefore omit some key contributions. Last, most of the research articles included in this study used a quantitative research design. More studies should be conducted using other methodologies, such as interviews and observations, to provide a more comprehensive picture of knowledge on P-ESDM.

## 6. Conclusions

In conclusion, this review shed light on the importance of the development and interaction between parents and children. Moreover, P-ESDM may provide an approach to improve child- and parent-related outcomes. Given our recommendations and limitations, further research is required to evaluate the correlations between the parent treatment fidelity of ESDM techniques and child outcomes. Furthermore, because the use of ESDM techniques differs between parents, a larger sample size for replicating the findings is necessary. In addition, the benefits and limitations of telehealth-delivered and P-ESDM interventions that include fathers should be considered.

## Figures and Tables

**Table 1:** Summary of child characteristics, type of study design, outcome measures, and results of parent fidelity of the included studies.

Study	Title 2			Study Design	Intervention Intensity and Duration	Outcome Measures		Parent Fidelity (Pretest→Posttest)
	N	Age (MOS)	Diagnosis			Child	Parent	
Abouzeid et al. (2020) [17]	10	18–45	Clinical diagnosis of ASD	Quasi-experimental one-group pretest–posttest	3 h/wk for 13 wk	No information	Satisfied or highly satisfied Positive perceptions of the relationship between the coach and their family	53% **→** 61%
Estes et al. (2014) [28]	49 (int.) 49 (TAU)	12–24	At risk of ASD	Randomized controlled trial	1 h/wk for 12 wk	No information	P-ESDM group reported no increase in parenting stress whereas community group increased. P-ESDM did not differ in their sense of competence compared to the community group	No scores but were analyzed using videotape
Hernandez-Ruiz (2018) [18]	3	30–36	At risk/clinical diagnosis of ASD	Quasi-experimental one-group pretest–posttest	0.5 h/wk for 10 wk	No information	Two of the mothers seemed to feel more competent Parent’s perception of their child’s social skills modified Decreasing from moderate distress to no distress The intervention was culturally and developmentally appropriate, enjoyable, and promoted interaction	No information
Hernandez-Ruiz (2020) [42]	1	48	Clinical diagnosis of ASD	Single-case, nonconcurrent alternating-treatment design	1 h/wk for 12 wk	Improvement in nonverbal responsiveness and initiation of joint attention	Parents seemed capable and found value in learning strategies from professionals that they could implement at home.	52–58% **→** 80% (linear)
Malucelli et al. (2021) [45]	9 (int.) 9 (TAU)	29–42	Clinical diagnosis of ASD	Randomized controlled trial	2 h/wk for 12 wk	Learning rate in different areas (except imitation) showed significant differences between two groups	Descriptive information was provided by the researchers regarding the high agreement of observation in 10-min videos of parent-child interaction	No information
Rogers et al. (2012) [15]	49 (int.) 49 (TAU)	12–24	At risk of ASD	Randomized controlled trial	1 h/wk for 12 wk	Improvement in both groups; no significant differences related to group assignment were noted	P-ESDM group did not exhibit significantly higher P-ESDM fidelity scores than the community treatment group. Parents receiving ESDM coaching exhibited stronger working relationships with their primary therapist than parents receiving community intervention	P-ESDM group had large effect size (0.57) compared with the community intervention group’s moderate effect size (0.37)
Rogers et al. (2019) [43]	45	12–30	At risk/clinical diagnosis of ASD	Randomized controlled trial	1.5 h/wk for 12 wk	Significant developmental acceleration; child outcomes did not differ by group	P-ESDM++ group exhibited significantly increased sensitivity and skill compared with the parents in the P-ESDM group Parents in the enhanced group exhibited significantly greater improvement in interaction skills than parents in the control group Parents were extremely satisfied with the intervention	3.4 **→** 3.8 (maximum of 5.0)
Vismara et al. (2012) [40]	9	16–38	Clinical diagnosis of ASD	Single-subject multiple-baseline design	1 h/wk for 12 wk	Children’s social-communicative behaviors increased significantly, as indicated by three independent data sources	Parental responsivity, affect, and achievement-oriented behaviors increased during intervention Parents gave positive ratings on a feasibility and acceptability questionnaire	2.62 **→** 4.29 (maximum of 5.0)
Vismara et al. (2013) [41]	8	18–45	Clinical diagnosis of ASD	Single-subject multiple- baseline design	1.5 h/wk for 12 wk	Use of functional verbal utterances, joint attention initiations(eye gaze alternation, i.e., giving, showing, or pointing), and receptive and expressive language increased during intervention and follow-up	Parent engagement increased during intervention and follow-up Parents gave positive ratings on a satisfaction survey	6 of 8 parents achieved fidelity (80%) on the ESDM fidelity scale during intervention and 7 of 8 parents during follow-up.
Vismara et al. (2016) [44]	14 (int.) 10 (TAU)	18–48	Clinical diagnosis of ASD	Randomized controlled trial	1.5 h/wk for 12 wk	No treatment effect for children’s social communication behaviors	Posttreatment, 36% of parents in the P-ESDM group compared with 20% of parents in the community-treated group achieved fidelity Parents in the P-ESDM group used the website more often and with higher satisfaction	0% **→** 36% of P-ESDM parents achieved fidelity
Vismara et al. (2019) [5]	4	18–48	fragile X syndrome	Single-subject multiple-baseline	1.5 h/wk for 12 wk	Standardized composite scores increased for all but one child	Parent fidelity increased Three out of four parents rated the intervention and coaching experience positively Parents also reported improvement of children’s understanding and usage of communicative gestures and words	below 4.00 **→** above 4.00 (maximum of 5.0)
Waddington et al. (2020b) [3]	5	23–59	At risk/clinical diagnosis of ASD	Quasi-experimental one-group pretest–posttest	1 h/wk for 12 wk	All mothers mentioned improvements in spoken and/or nonverbal communication	All five mothers gave the intervention positive ratings through the Treatment Acceptability Rating Scale-Revised. Four mothers commented on the positive personal qualities and knowledge of the trainer.	No information (only reported four of the mothers improved)
Zhou et al. (2018) [12]	23 (int.) 20 (TAU)	18–30	Clinical diagnosis of ASD	Nonrandomized controlled trial	1.5 h/wk for 26 wk	Neither group exhibited significant change in ASD severity, but the P-ESDM group exhibited greater improvement in social affect	Parents in the P-ESDM group experienced decreased parenting stress, but those in the community intervention group exhibited an opposite trend.	No information

MOS months, Int. intervention group, TAU treatment as usual group.

## Data Availability

Not applicable.
